# Examining the impact of premenstrual dysphoric disorder (PMDD) on life and relationship quality: An online cross-sectional survey study

**DOI:** 10.1371/journal.pone.0322314

**Published:** 2025-04-23

**Authors:** Sophie Hodgetts, Aaron Kinghorn

**Affiliations:** 1 Department of Psychology, Durham University, Durham, United Kingdom; 2 International Association for Premenstrual Disorders, Board of Directors, Durham, United Kingdom; University of the Basque Country UPV / EHU, SPAIN

## Abstract

Little is known regarding the impact of premenstrual dysphoric disorder (PMDD) on specific aspects of life quality within the home, such as spousal/partner relationships. Moreover, the impact of PMDD on the partners of those with the condition has not been investigated. Therefore, the present study examined the ways in which PMDD can affect the perceived life and relationship quality of both those with the condition, and their partners. Across two studies, cross-sectional survey methods were used to compare perceived quality of life and relationship quality between PMDD patients (n = 216) and controls (n = 187), and between PMDD partners (n = 92) and controls (n = 59). In both PMDD patients and their partners, perceived quality of life was lower across most domains compared to controls. Additionally, both PMDD patients and their partners reported lower relationship quality compared to controls, for all domains except love and commitment. Our findings indicate that PMDD has a wide-ranging impact on both the affected individual and their partner. Future clinical research should aim to develop PMDD-specific interventions that support both the person with PMDD and their partner.

## 1. Introduction

Premenstrual symptoms (PMS) are common, affecting an estimated 80%-90% of those with a naturally occurring menstrual cycle [1–3]. Typical symptoms include affective lability (e.g., mood swings, irritability, low mood), cognitive disturbances (e.g., memory impairment, executive dysfunction), and physical discomfort (e.g., cramping, bloating, pain in breasts) [4]. However, substantial individual differences exist and for most people these symptoms do not reach clinically significant levels of severity [5]. Nevertheless, approximately 1.6% of those with natural menstrual cycles experience symptoms that meet the diagnostic criteria for premenstrual dysphoric disorder (PMDD) [6]. PMDD is a potentially disabling condition characterised by the cyclical recurrence of clinically distressing and/or impairing affective, psychological, and physical symptoms during the luteal/premenstrual cycle phase (i.e., two weeks before menstruation onset, [7–10]. Typical affective symptoms include irritability/anger, rejection sensitivity, depressed mood/anhedonia, feelings of worthlessness/guilt, anxiety, and suicidal ideation. Patients may also experience cognitive impairment (e.g., memory impairment, executive dysfunction). Diagnosis of PMDD according to the DSM-V requires the presence of at least five symptoms, of which one must be affective, during the luteal phase, as confirmed by prospective cycle tracking and symptoms monitoring. While PMDD affects similar domains as PMS, it differs in severity and in the degree of impairment caused, with psychological/psychiatric symptoms being particularly prominent in PMDD.

Evidence to date suggests that PMDD has a significant negative impact on the individual’s quality of life and daily functioning, particularly during the symptomatic phase [1]. For example, greater PMDD symptom severity has been associated with a reduction in quality-adjusted life years [11], increased absenteeism and decreased productivity [12–15], increased use of multiple health services [12,16], reduced school efficiency [13,17], and reduced participation in social activities [12,13,17,18]. Indeed, the impact of PMDD on quality of life is likely comparable to that seen in other psychiatric conditions (e.g., dysthymia and major depressive disorder, [8]).

In contrast little is known about the potential for PMDD to impact specific aspects of life quality and functioning within the home, specifically regarding interpersonal relationship quality with spouses/partners [7,8]. The limited evidence base suggests that PMDD (which is sometimes referred to, incorrectly, as “severe PMS” in previous studies) is characterised by functional impairment within the home [14] and reduced marital relationship satisfaction [19,20]. Such findings are important, as evidence from other psychiatric populations indicates that high quality interpersonal relationships (including marital and other romantic relationships) can act as a protective factor against the impact of psychiatric symptoms [21,22]. However, the studies of PMDD effects on interpersonal relationships are subject to limitation. Firstly, most of these studies either conflate PMDD and PMS or use refer to PMDD as “severe PMS”. Given that the formal diagnostic criteria for PMDD is stringent and designed to differentiate PMDD from (severe) PMS, it is possible that the true impact of PMDD has been underestimated in these studies. Secondly, studies to date have not considered the impact of PMDD on non-marital relationships (e.g., cohabiting long-term partners) or non-heteronormative relationships (e.g., same-sex partnerships, transgender PMDD patients/partners). Therefore, further research that accounts for these limitations is necessary considering evidence suggesting that interpersonal conflict (e.g., conflict between PMDD patients and their partners) may result in increased PMDD symptom severity [23].

While investigating the impact of PMDD on interpersonal relationships is important with regards to PMDD patients themselves, it is also important to consider the impact of PMDD on their partners (e.g., husband, wife, spouse etc). These individuals typically sit at the intersection of being a partner and a familial caregiver for their PMDD-affected partner. Studies in other psychiatric populations (e.g., depression, bipolar disorder, and schizophrenia) have shown that familial caregivers (including partners) are at higher risk of developing physical [24] and/or mental illness [25–27]) compared to the general population. These risks are independent of other stressors, and further increased in instances of limited social or professional support [26]. In contrast, access to psychosocial support (e.g., psychoeducation, group-based talking therapies, professional nursing support) for familial caregivers of individuals with a mental illness is associated with a reduction in risk to physical/mental health for the caregiver [28–31] and in turn, increased caregiving capacity, ultimately resulting in a better quality of life for all involved, including the patients themselves [32,33]. Such studies are needed for PMDD, as the ability of the partner to provide care and/or negate interpersonal conflict may be compromised if they also become unwell. In addition, given the relatively unknown nature of PMDD compared to other psychiatric diagnoses, social support for the families of patients is likely [7]. Therefore, examining how PMDD affects partners regarding their perceived life and relationship quality, is likely to provide valuable insight concerning their support needs, which will in turn inform caregiver-focused support, resulting in benefits for both the caregiver and their PMDD-affected partner.

In sum, while evidence to date suggests that PMDD can have a significant impact on life quality and functioning, no studies have investigated the ways in which PMDD can affect interpersonal relationships and functioning within the home. Moreover, knowledge is lacking regarding the impact PMDD may have on the partners of those with the condition.. Such studies are needed to provide insight regarding the support needs of both parties [7]. Therefore, we designed two studies to investigate the specific aspects of life and relationship quality that may be affected by PMDD in both those with the condition and their partners. In Study 1, the effect of PMDD was investigated by comparing several outcome measures between those with the condition and a control group (i.e., those who have a naturally occurring menstrual cycle but are not diagnosed with PMDD). In Study 2, the effect of PMDD on the quality of life and relationships in people who are partners of someone with PMDD was investigated by comparing outcomes between them and a control group (i.e., people who are partners of someone with a naturally occurring menstrual cycle who do not have PMDD). It was hypothesised that across both studies, the presence of PMDD would be associated with lower life quality, and lower perceived relationship quality.

## 2. Study 1

### 2.1. Materials and methods

#### 2.1.1. Participants.

Four hundred and three participants (from a total of 1061) with a mean age of 31.34 years (SD = 8.84) were included in the analysis. Participants were excluded if they met one of more of the following criteria: did not provide full consent, had missing responses (e.g., skipped questions), were not in a relationship (e.g., single, casually dating), reported having no periods and/or were unsure of their current menstrual cycle phase. Participants were recruited using community sampling, responding to an online study advert targeting people with menstrual cycles. This included public-facing posts on social media (e.g., Facebook, X/Twitter), and targeted advertising via the International Association for Premenstrual Disorders (IAPMD) webpage and social media to recruit those with PMDD. Additional control participants were recruited from the undergraduate student population of the first author’s institution. No incentives were offered for completing the study. All data was collected between November 2021 and December 2022. Participants provided informed consent using a digital consent form; after reading the information sheet, participants navigated to the consent form on the following screen and indicated their consent to participate via a tick box. Participants were then able to access and complete each of the questionnaires listed in 2.1.2. Ethics approval (for both studies) was obtained from the Psychology Research Ethics Committee of the first author’s institution.

Participants were assigned to either the PMDD group (n = 216) or the control group (n = 187) based on their scores from the Premenstrual Symptoms Screening Tool (PSST, see 2.1.2). It should be noted that approximately 74% of the control group met the PSST criteria for PMS, and some had previously been formally diagnosed PMDD or suspected they may have PMDD (approx. 35%). However, as they did not meet the threshold for PMDD at the time of testing according to their PSST scores they were placed in the control group. We posit that this results in a control group that more accurately reflects the non-PMDD population, which in turn allows for meaningful comparisons to be made that can be generalised beyond the current study’s sample. Moreover, splitting the groups into samples of PMDD, PMS, suspected PMDD, and no symptoms would result in unbalanced, small samples, which would render any differences difficult to interpret and unlikely to be meaningful. Participants were further divided according to their self-reported cycle phase (i.e., menstrual phase, follicular phase, luteal phase, premenstrual phase). A factorial ANOVA revealed a significant group effect on age (*F*_(1, 395)_ = 17.98, *p* <.001, η_p_^2^ =.044), with the PMDD group being older than the control group compared to the control group; however, we note that this is a small-medium effect size, suggesting that the practical impact of age on our DVs is minimal and other factors likely contribute to the variance more than age. It is likely that the difference in age is due to the length of time associated with the PMDD diagnostic process (e.g., an average delay of 20 years was recently reported by [34]). Demographic information for the whole sample and their partners is shown in Table 1.

**Table 1:** Age (mean ± standard deviation), sex, gender (of respondent and their partner), and relationship type.

	Premenstrual dysphoric disorder				Control			
**Menstrual** **N = 51**	**Follicular** **N = 50**	**Luteal** **N = 77**	**Pre-menstrual** **N = 38**	**Menstrual** **N = 39**	**Follicular** **N = 50**	**Luteal** **N = 51**	**Pre-menstrual** **N = 47**
**Respondent age**	33.39 ± 7.11	33.78 ± 8.19	32.48 ±7.79	31.18 ± 8.89	28.89 ± 11.01	29.94 ± 7.83	30.29 ± 8.84	27.78 ± 9.99
**Respondent gender**	50 Female 1 Non-binary	48 Female 2 Non-binary	74 Female 3 Non-binary	37 Female 1 Non-binary	37 Female 1 Male 1 Non-binary	44 Female 1 Male 5 Non-binary	48 Female 3 Non-binary	45 Female 2 Non-binary
**Partner** **age**	35.70 ± 9.05	36.30 ± 7.58	35.1± 8.65	33.50± 10.87	30.07 ± 9.14	31.20± 8.67	33.58± 11.60	30.11± 12.74
**Partner** **sex**	2 Female 48 Male 1 missing	4 Female 46 Male	6 Female 70 Male 1 missing	4 Female 34 Male	4 Female 35 Male	4 Female 46 Male	4 Female 47 Male	3 Female 44 Male
**Partner gender**	1 Female 50 Male	4 Female 46 Male	5 Female 70 Male 2 Non-binary	4 Female 34 Male	2 Female 37 Male	1 Female 46 Male 3 Non-binary	3 Female 46 Male 2 Non-binary	2 Female 44 Male 1 Non binary
**Relationship type**	14 in a relationship living separately 12 in a relationship and cohabiting 24 married and cohabiting 1 other	9 in a relationship living separately 17 in a relationship and cohabiting 1 married living separately 24 married and cohabiting 1 other	24 in a relationship living separately 22 in a relationship and cohabiting 1 married living separately 30 married and cohabiting	14 in a relationship living separately 15 in a relationship and cohabiting 9 married and cohabiting	15 in a relationship living separately 11 in a relationship and cohabiting 12 married and cohabiting 1 other	5 in a relationship living separately 11 in a relationship and cohabiting 12 married living separately 22 married and cohabiting	18 in a relationship living separately 13 in a relationship and cohabiting 20 married and cohabiting	27 in a relationship living separately 13 in a relationship and cohabiting 7 married and cohabiting

NB. ‘other’ relationship types included long-distance relationships and engaged couples

#### 2.1.2. Questionnaires.

***Relationship and menstrual cycle screening:*** All participants were asked to provide their current relationship status from a series of options: In a relationship (not married, living apart), in a relationship (not married, living together), Married but not living together, married and living together, or other. Participants who selected “other” were asked to provide details using free text boxes. Participants were then asked to provide demographic information for themselves and their partner (i.e., age, sex, gender, ethnicity, education history, current employment).

Menstrual cycle length and regularity was assessed using self-report screening questions adapted from previous studies [35] and following current guidance for studies in which menstrual cycle phase is not the primary variable of interest [7,36]. Participants were asked to indicate how regular their menstrual cycle is (“Very regular (period starts within 3-4 days of its due date)”, “Regular (period starts within 5-7 days of its due date)”, “Usually irregular”, “Always irregular”, “No periods”, “Unsure”), their typical cycle length (< 21 days, 22–25 days, 26–31 days, 32–39 days, 40–50 days, > 50 days, “Too irregular to estimate”, “unsure”), the number of days since their the start of their more recent period, and their current cycle phase (“Currently menstruating”, “ Follicular phase (i.e., most recent period started 10–14 days ago), “Luteal phase (i.e., most recent period started more than 15 days ago and next period has not started yet)”, “Pre-menstrual (i.e., next period is about to start/is due in the next few days)”, “Unsure”. Participants were excluded if they had no periods/were unsure of their current cycle phase, meaning most participants reported a regular or very regular cycle (approx. 82%).

***Premenstrual Symptoms Screening Tool (PSST):*** The PSST [37] is a 19-item questionnaire that uses the categorical DSM criteria associated with PMDD and PMS as a symptom rating scale, with degrees of severity. It contains items covering the emotional and physical symptoms associated with PMDD/PMS, as well as items assessing the impact of symptoms on daily function. Each item is rated on a 4-point scale indicative of symptom severity (‘not at all’ to ‘severe’). PMDD is indicated if participants respond ‘severe’ to at least one item in the emotional category, ‘moderate’ to ‘severe’ in at least four other items, and the impact of symptoms is ‘severe’ in at least one area of daily function. PMS is indicated if participants respond ‘moderate’ or ‘severe’ to at least one item in the emotional category, ‘moderate’ to ‘severe’ in at least four other items, and the impact of symptoms is ‘moderate’ or ‘severe’ in at least one area of daily function. Participants who do not meet any of these criteria are classified as having no premenstrual symptoms. In the present study, we used PSST scores and self-reported diagnoses to create participant groups.

***World Health Organisation Quality of Life Questionnaire - Brief Version (WHOQoL-BREF):*** The WHOQoL-BREF [38] is a 26-item questionnaire comprised of four subscales designed to assess life quality in four domains: physical health, psychological health, social relationships, and environmental health, with a higher score indicating higher quality of life in that domain. All items are rated using a 5-point Likert scale and all scores were transformed to a 0–100 scale. Scores from each subscale, as opposed to the total score, were included in the analysis to fully identify the specific domains in which PMDD impacted quality of life.

***Perceived Relationship Quality Component Scale (PRQC):*** The PRQC [39] is an 18-item questionnaire comprised of six subscales designed to assess quality across different aspects of romantic relationships including relationship satisfaction, commitment, trust, intimacy, passion, and love. All items are rated using a 7-point Likert scale, and scores for each subscale were calculated by averaging across the relevant items with a higher score indicating higher perceived quality. Scores from each subscale, as opposed to the total score, were included in the analysis to fully identify the specific aspects of relationships that may be affected by PMDD.

#### 2.1.3. Data analysis.

All data was submitted to a multivariate analysis of variance (MANOVA) using SPSS 27. A MANOVA was chosen as this allows for simultaneous, multiple comparisons across the dependent variables (i.e., individual subscales), which reduces the risk of Type 1 error by providing a composite outcome. Where post-hoc tests were needed to explore effects on individual dependent variables following a significant MANOVA result, Bonferroni corrections were applied to adjust for multiple comparisons. Age was not included as a covariate in the analysis, as it is likely that the higher age in the PMDD group is due to time taken to obtain a diagnosis and therefore, age is more likely to be a collider as opposed to a confounding variable. As such, controlling for age would render the results less generalisable to the wider PMDD population. For PMDD patients/controls, each MANOVA included group (PMDD, control) and cycle phase (menstrual, follicular, luteal, pre-menstrual) as a between subjects’ factors and subscale scores from each questionnaire as the dependent variables.

## 3. Results

### 3.1. World Health Organisation quality of life brief version

**Fig 1:**
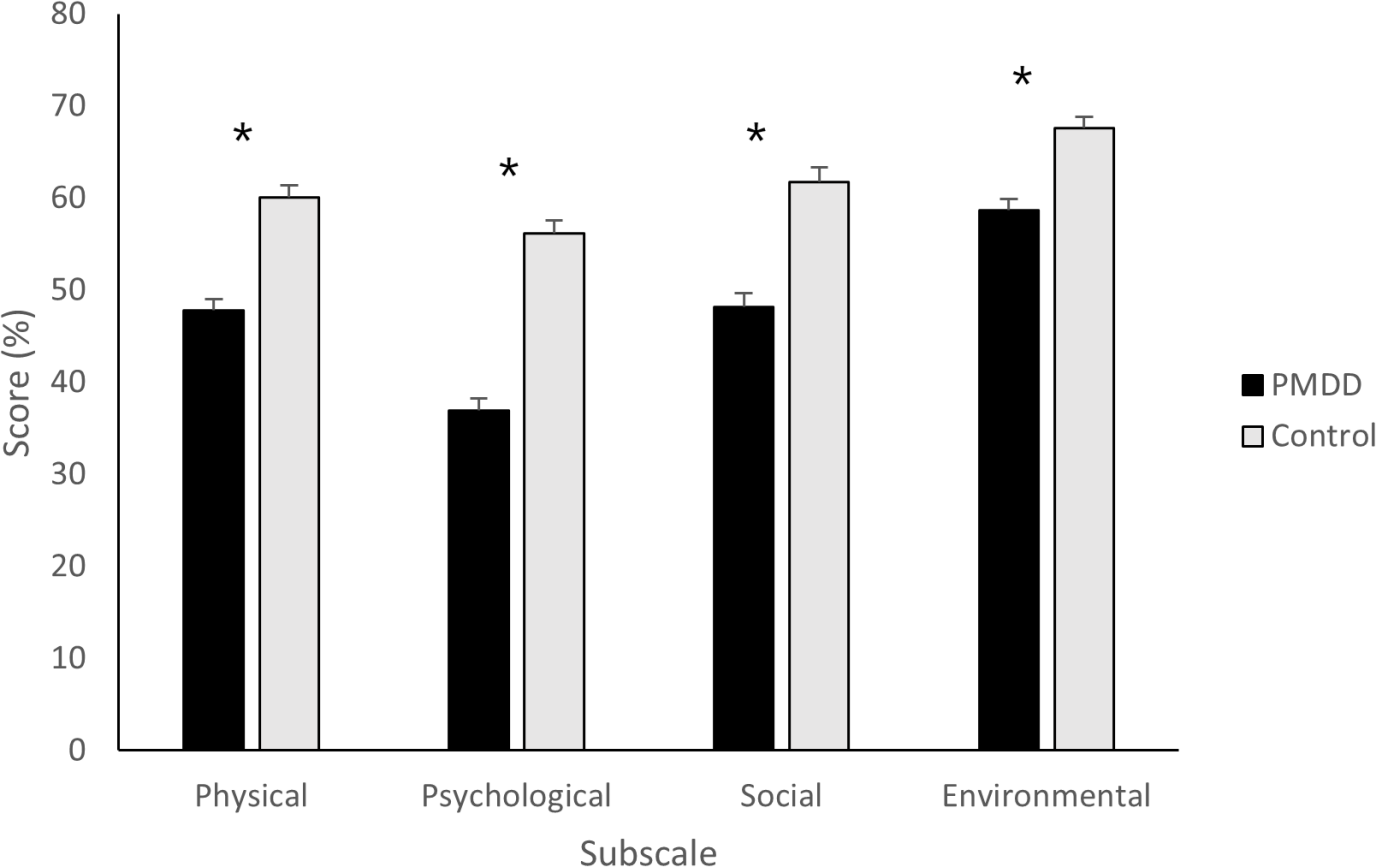
Mean WHOQoL-BREF scores and standard error means for each subscale according to group (PMDD, control) for each subscale. Higher scores indicate a higher perceived quality of life in the respective domain (* = p <.001).

Box’s M was non-significant (*p* =.624), and so equal covariance matrices are assumed. The overall multivariate MANOVA results for the WHOQoL-BREF subscales revealed a significant effect of group with a large effect size (Pillai’s Trace: *V* =.209, *F*_(4, 392)_ = 25.854, *p <*.001, η_p_^2^ =.209), indicating an overall difference between the PMDD and control groups. This was supported by follow-up univariate tests, which showed significant differences between the groups for all four individual subscales (all *F* > 21.871, all *p* <.001). Bonferroni corrected pairwise comparisons showed that the PMDD group scored significantly lower than the non-PMDD group, for each individual subscale (all *p* <.001, Fig 1).

The MANOVA also revealed a significant effect of cycle phase (Pillai’s Trace: *V* =.067, *F*_(12, 1182)_ = 2.243, *p* <.05, η_p_^2^ =.022). However, follow-up univariate tests indicated significant effects of cycle phase for the physical (*F*_(3,395)_ = 4.135, *p* <.05, η_p_^2^ =.03) and social (*F*_(3,395)_ = 3.632, *p* <.05, η_p_^2=^.027) subscales only. Moreover, both the MANOVA and follow-up univariate tests yielded small-medium effect sizes, suggesting that the impact of cycle phase was small compared to other influences on these scores. Bonferroni corrected pairwise comparisons showed that for the physical scale, there was a significant difference between the follicular phase and both the menstrual (*p* =.05) and pre-menstrual phases (*p* =.012). In both cases, the follicular group scored significantly higher (*M* = 58.07, *SE* = 1.806) compared to both the menstrual (*M* = 51.09, *SE* = 1.921) than the pre-menstrual group (*M* = 49.79, SE = 1.97).

### 3.2. Perceived Relationship Quality Component Scale

**Fig 2:**
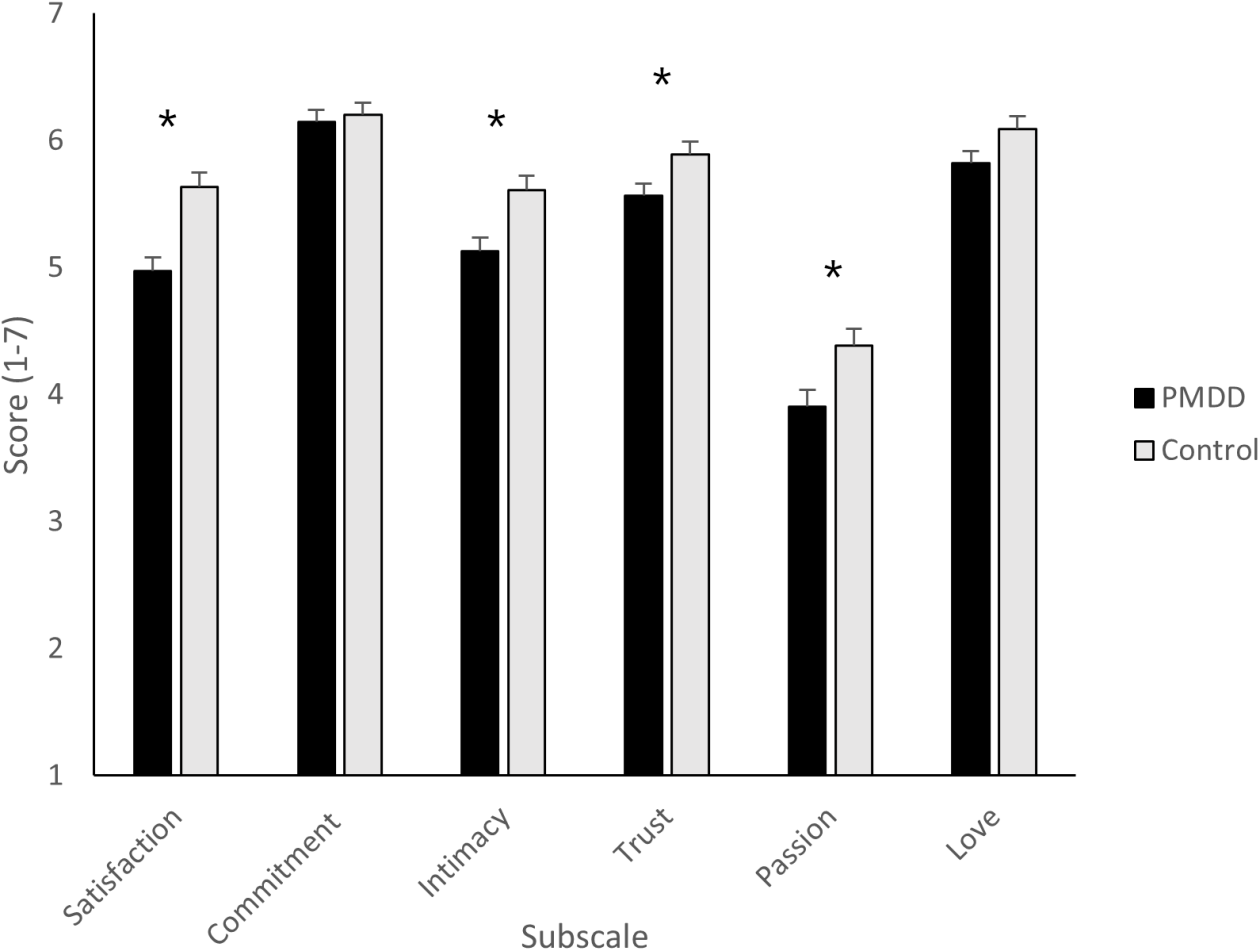
Mean PRQC scores and standard error means for each subscale according to group (PMDD, control). Higher scores indicate greater levels of the respective component (* = p <.02).

Box’s M was significant (*p* <.001), meaning the covariance matrices cannot be assumed as equal. The MANOVA revealed a significant effect of group and a medium-large effect size (Pillai’s Trace: *V* =.074, *F*_(6, 390)_ =.074, *p* <.001, η_p_^2^ =.964), indicating an overall difference between the PMDD and control groups. Examination of the follow-up univariate tests revealed a significant effect of group for relationship satisfaction (*F*_(1,395)_ = 18.66, *p* <.001, η_p_^2^ =.045), intimacy (*F*_(1,395)_ = 9.57, *p* <.002, η_p_^2^ =.024), trust (*F*_(1,395)_ = 5.70, *p* <.05, η_p_^2^ =.014), and passion (*F*_(1,395)_ =6.704, *p* <.01, η_p_^2^ =.017), all with small-medium effect sizes. Bonferroni corrected pairwise comparisons showed that the PMDD groups scored significantly lower than the non-PMDD group for each of these subscales (all *p* <.02). No effect of group was found for commitment or love (both *F* < 3.62, both *p* >.058, see Fig 2). There was no effect of cycle phase (Pillai’s Trace: *V* =.062, *F*_(18, 1176)_ = 1.398, *p* =.138), and no interaction effect (Pillai’s Trace: *V* =.054, *F*_(18, 390)_ = 1.202, *p* =.251).

## 4. Study 2

### 4.1. Materials and methods

#### 4.1.1. Participants.

One hundred and fifty-one participants (from a total of 528) with a mean age of 33.85 years (SD = 10.28) were included in the analysis. The consent procedure, and exclusion criteria were the same as in Study 1. The recruitment method was also largely the same as Study 1, with additional advertising done via PMDD Partner Support Groups, convened by IAPMD and facilitated by one of the authors (AK). All data was collected between November 2021 and December 2022.

Participants were asked to indicate whether their partner had PMDD, thus creating two groups: the PMDD partners group (n = 92) and the control group (n = 59). It should be noted that the PSST was not administered in this study, participants were included based on a self-report of their partners’ PMDD only. Participants who indicated that their partner had PMDD were also asked when the diagnosis occurred with respect to the relationship (e.g., before or during the relationship), and if their partner was currently symptomatic. It should be noted that approximately 33% of the control group reported that their partners had another mental health condition (e.g., depression, anxiety). However, as the aim of this study was to investigate the effect of PMDD specifically, we included these participants in the control groupAn independent samples t-test revealed that, like the PMDD patients, the PMDD partners were significantly older than the control group (*t*_(108.98)_ = 6.18, *p* <.001). Demographic information for the whole sample and their partners is shown in Table 2.

**Table 2:** Age (mean ± standard deviation), sex, gender (of respondent and their partner), and relationship type.

	PMDD partners N = 92	Control partners N = 59
**Respondent age**	37.66 ± 8.99	27.89 ± 10.04
**Respondent gender**	14 Female 76 Male 1 Non-binary 1 Other	15 Female 40 Male 4 Non-binary
**Respondent sex**	15 Female 76 Male 1 Other	20 Female 39 Male
**Partner age**	35.68 ± 7.26	27.36 ± 9.04
**Partner gender**	89 Female 3 Non-binary	56 Female 3 Non-binary
**Relationship type**	13 in a relationship living separately 3 married living separately 44 married and cohabiting 30 in a relationship and cohabiting 2 other	26 in a relationship living separately 17 married and cohabiting 1 married living separately 15 in a relationship and cohabiting

NB. ‘other’ relationship types included cohabiting but “recently separated” couples, included as they still labelled the other as a partner

#### 4.2.1. Questionnaires.

Relationship screening questions and the PRQC were administered as in Study 1.

***Adult Carer’s Quality of Life Questionnaire (AC-QoL):*** The AC-QoL [40] was used instead of the WHO-QoL BREF to assess partners’ quality of life. This questionnaire consists of 40 items designed to assess life quality in adult carers across eight domains: support for caring, caring choice, caring stress, money matters, personal growth, sense of value, ability to care, and carer satisfaction. All items are rated using a 4-point Likert scale and summed to provide a score of 0–15 for each subscale. While participants in this study may not identify themselves as carers, this questionnaire was chosen as it contains items that directly relate to the potential issues faced by the partners of someone with PMDD (e.g., *“I am happy with the professional support provided to me.”*, *“I have a good relationship with the person I am caring for.”*). Moreover, for the purposes of this study, the questionnaire instructions were clarified accordingly for participants as follows: “*This questionnaire asks you about different aspects of your life as a carer/partner of someone with premenstrual dysphoric disorder/a menstrual cycle (deleted accordingly). Although questionnaire is used for carers, please answer it in as much as it applies to yourself as we will compare your responses to those who care for someone with premenstrual dysphoric disorder/someone with a menstrual cycle (deleted accordingly).”*

#### 4.1.3. Data analysis.

All data was submitted to a MANOVA, which included group (partner of someone with PMDD, partner of someone without PMDD) as a between-subjects factor and scores from each questionnaire as the dependent variable. Cycle phase was not included in this analysis, as most respondents were unable to provide this information at the time of testing.

## 5. Results

### 5.1. Adult Carer’s Quality of Life Questionnaire (AC-QoL)

**Fig 3:**
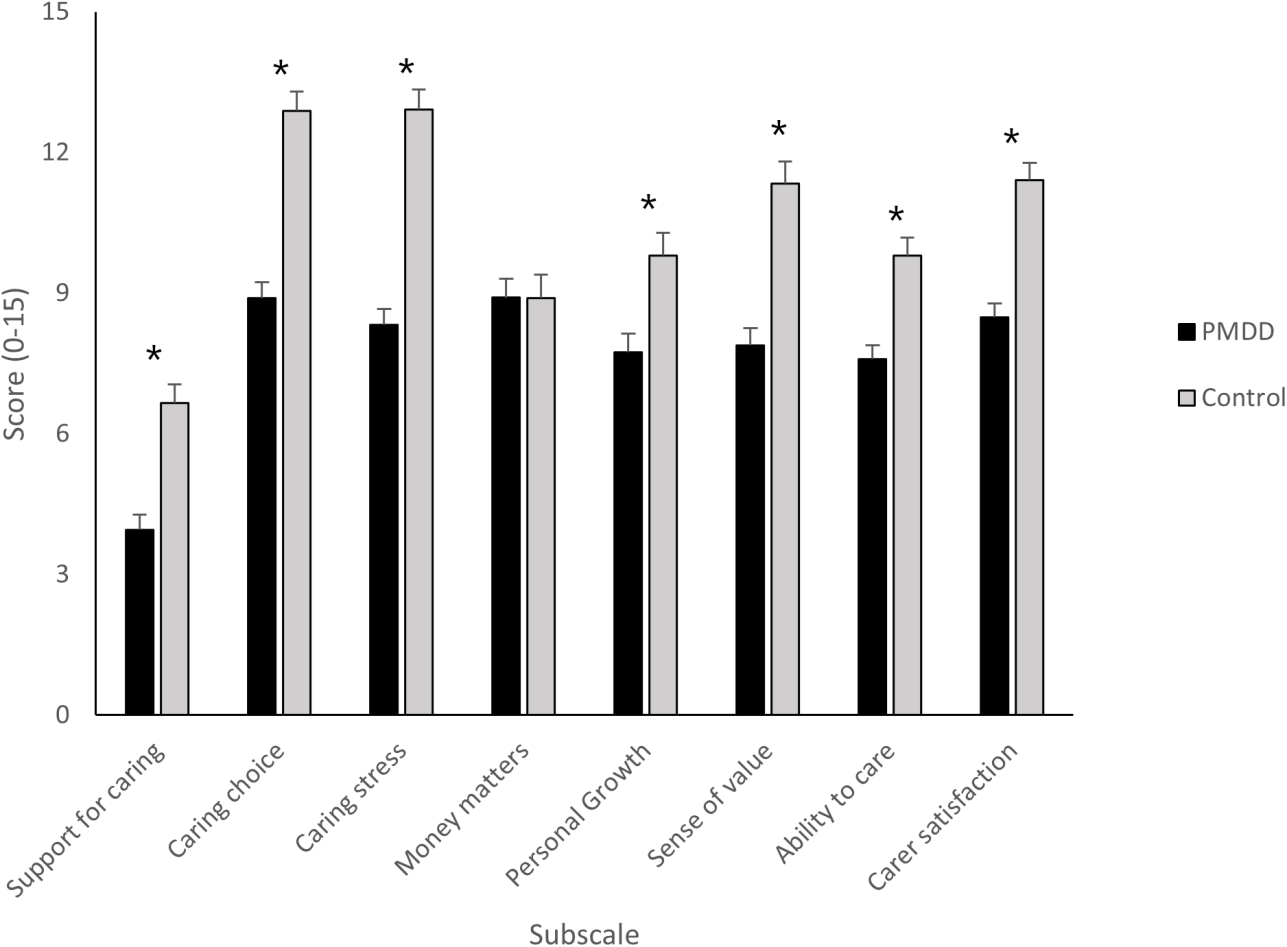
Means scores and standard error means according to group (PMDD partners, control partners) for each subscale of the AC-QoL. Higher scores indicate a higher quality of life in the respective domain (* = p <.01).

Box’s M was significant (p <.05) meaning the covariance matrices cannot be assumed as equal. The MANOVA results for the AC-QoL subscales revealed a significant effect of group and a large effect size (Pillai’s Trace: *V* =.040, *F*_(8, 142)_ = 11.82, *p* <.001, η_p_^2^ =.400, indicating an overall difference between partners of PMDD patients and control partners. Examination of the follow-up univariate tests revealed a significant effect of group for caring support, caring choice, caring stress, personal growth, sense of value, ability to care, and satisfaction with caring (all *F* > 10.77, all *p* <.01, see Fig 3). Only the financial implications of caring subscale yielded a non-significant difference between the groups and a small effect size (*F*_(1, 151)_ =.001, *p* =.995, η_p_^2^ <.001).

### 5.2. Perceived Relationship Quality Component Scale (PRQC)

**Fig 4:**
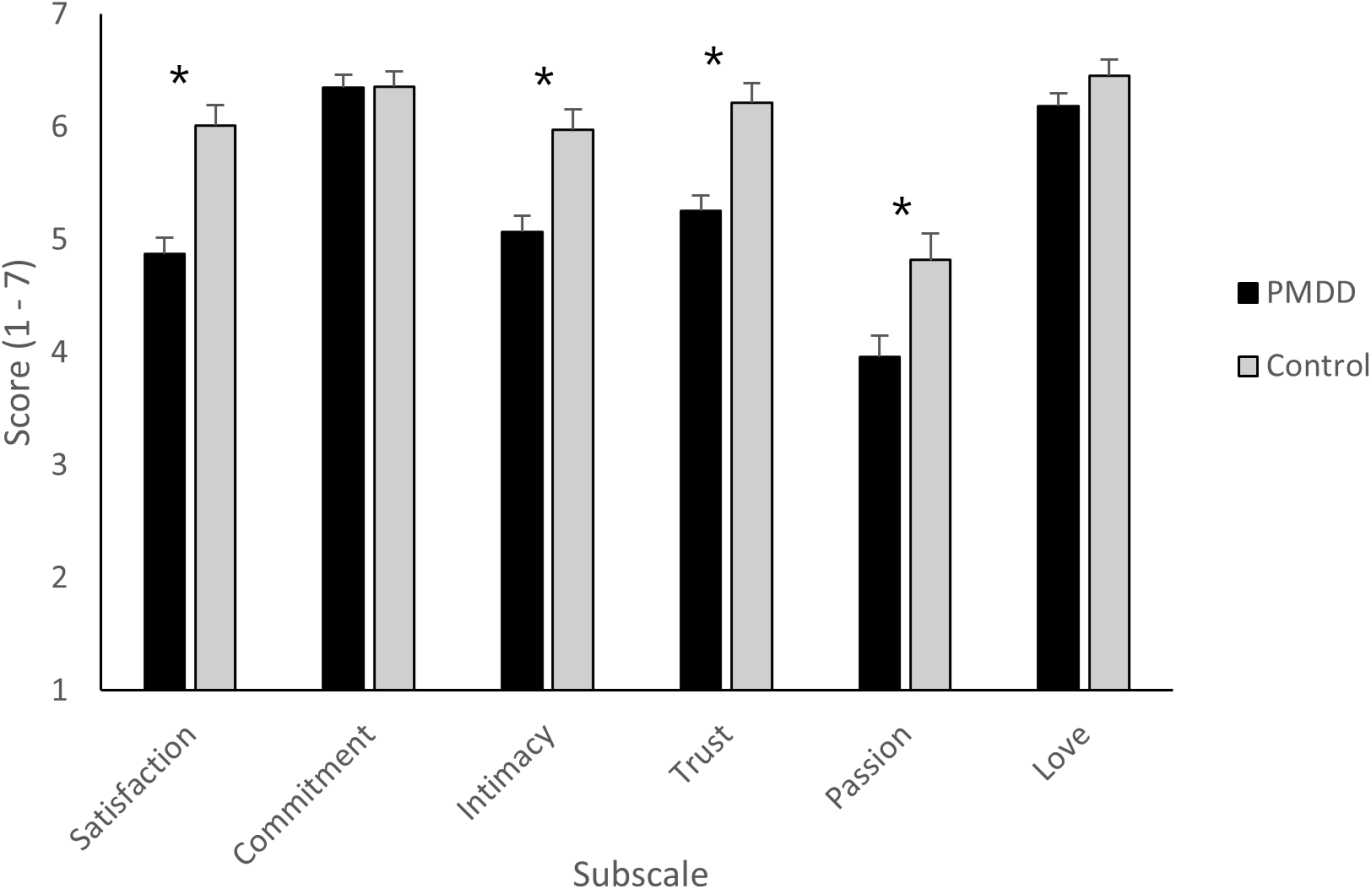
Mean scores and standard error means according to group (PMDD, control) for each subscale of the PRQC. Higher scores indicate greater levels of the respective component (* = p <.005).

Box’s M was significant (*p* <.001) meaning the covariance matrices cannot be assumed as equal. The MANOVA results for the PRQC subscales revealed a significant effect of group and a large effect size (Pillai’s Trace: *V* =.216, *F*_(6, 144)_ = 6.62, *p* <.001, η_p_^2^ =.216, indicating an overall difference between the PMDD partners and control partners. Examination of the follow-up univariate tests revealed a significant effect of group for relationship satisfaction (*F*_(1,149)_ = 23.04, *p* <.001, η_p_^2^ =.134), intimacy (*F*_(1,149)_ = 15.93, *p* <.001, η_p_^2^ =.097), trust (*F*_(1,149)_ = 18.70 *p* <.001. η_p_^2^ =.111), and passion (*F*_(1,149)_ = 8.25, *p* <.01, η_p_^2^ =.052), all with medium-large effect sizes. Bonferroni corrected pairwise comparisons showed that the PMDD partners scored significantly lower than the non-PMDD group for each of these subscales (all *p* <.005). No effect of group was found for commitment or love (both *F* < 2.37, both *p* >.126, see Fig 4).

## 6. Discussion

The present studies aimed to investigate the ways in which PMDD can affect the quality of life and relationships in both those with the condition and their partners. Study 1 revealed that having PMDD was associated with significantly lower life quality compared to controls, in all four of the assessed domains (physical, psychological, social, environment). There was a significant effect of cycle phase however, and both PMDD and non-PMDD groups reported lower scores in the physical domain during the menstrual and premenstrual phases, relative to the follicular phase. It seems likely that this is due to a similar pattern of physical symptoms during the pre- and menstrual phases in both groups (e.g., cramping, bloating, discomfort). Perceived relationship quality was also significantly lower in the PMDD group, for all domains except love and commitment. Study 2 revealed that PMDD partners also experienced lower quality of life compared to controls in most of the assessed domains (support for caring, caring choice, caring stress, personal growth, sense of value, ability to care, carer satisfaction); only the financial domain was associated with no difference between the groups. Regarding perceived relationship quality, Study 2 revealed a similar pattern of results to those seen in Study 1; that is, perceived relationship quality was significantly lower for the PMDD partners, for all domains except love and commitment.

In line with the hypotheses and previous findings [11,17,18], PMDD was associated with lower quality of life in all the domains measured. This finding was consistent between cycle phases for the psychological, environmental, and social domains, suggesting that the effect of PMDD on life quality in these domains is maintained beyond the symptomatic phase (i.e., luteal/premenstrual). Examining each subscale yields further insight regarding the implications of this finding. For example, as the psychological subscale focuses on patients’ cognitions, this suggests that consistent non-pharmacological support is needed. Additionally, examining the social subscale, which focuses on the social support available to patients, reveals that psychoeducation for the families/partners of PMDD patients may be needed. Finally, as the environmental scale focuses on the physical conditions in which the patient lives, this suggests that more community-based support for PMDD is needed.

Taken together, these findings suggest that consistent, non-pharmacological support for PMDD is needed. The medium-large effect sizes that were consistently yielded in our studies when comparing the PMDD and non-PMDD groups further emphasise this need as they indicate that adapting clinical intervention to support patients’ interpersonal relationships (e.g., relationship quality) would have clinically significant impact. Indeed, family-focused therapy (FFT), comprised of psychoeducation, communication-enhancement, and problem-solving skills training, may provide support for both patients and their families in each of the affected domains. However, while evidence supporting the use of FFT exists for some disorders (e.g., bipolar disorder, [41]), non-pharmacological interventions for PMDD lack supporting evidence [6]. It is also possible that couples’ interventions would be beneficial for those affected by PMDD. Indeed, a systematic review [42] reports that couples’ interventions (including cognitive behavioural therapy and relationship counselling) for chronic physical health conditions generally yield better physical and psychological outcomes compared to patient-only interventions. Successful interventions focused on skill development for managing complex emotions, as well as elements of counselling and cognitive behavioural therapy. However, such interventions been applied to or studied in a limited number of conditions, and typically focus on physical illnesses as opposed to mental illnesses. Thus, the present study supports the need for future studies to investigate the effectiveness of family and/or couples’ interventions for PMDD, with couples involved in the development of the intervention and with partner outcomes routinely acknowledged alongside patient outcomes.

Lower quality of life was also present in the PMDD partners compared to controls in all domains, except for the financial domain. We note that medium-large effect sizes were present across all comparisons where a significant difference was found, indicating that our findings have practical implications. Specifically, this suggests that tailored support accounting for the needs of PMDD partners is lacking in current practice. Indeed, the mean score in the “support for caring” domain was in the low quality of life range (0–5), indicative of significant problems and/or difficulties [43]. This subscale assesses the extent of practical and professional support that carers receive, as well as the extent of support available to them for their own emotional/psychological needs. This finding is important as it suggests that the support needs of PMDD partners are currently going unmet, potentially placing them at risk of developing mental and/or physical illness themselves [26]. Furthermore, it is possible that difficulties in this domain underpin the differences in scores for other domains, as previous research suggests that access to support for carers is associated with increased caregiving capacity, ability, and satisfaction [32,33]. Therefore, future research is needed to develop evidence-based support specifically for the partners and informal carers of those with PMDD. For example, evidence from other disorders has shown that peer support groups (e.g., for informal carers, families, partners) can have a positive outcome on informal carers’ mental health and support them when faced with caring-related challenges [44,45]. Finally, the lack of a difference between the groups for the financial domain warrants discussion. Both groups scored in the mid-range for this domain, suggesting that neither group experienced a high or low quality of life. Therefore, it seems likely that this finding reflects the universally negative effects of the current “cost-of-living crisis”, characterised by a global decrease in disposable incomes and increased energy costs [46].

Concerning relationship quality, both participants with PMDD and their partners perceived less intimacy, trust, and passion in their relationship compared to controls (with medium-large effect sizes), while no differences were found for love and commitment. Examining the items for intimacy, trust, and passion, it is possible the group differences are driven by the cyclical nature of PMDD symptoms, and the behaviour associated with the symptoms. For example, items on the trust subscale rely on the respondents’ perception of the partner, and as such, responses are likely susceptible to state effects, such as anxiety [47]. In contrast, items on the love and commitment scales refer to emotions that are not typically attenuated by anxiety but have been shown to reduce anxiety and other negative emotions [48]. It is also noteworthy that the love and commitment scores are comparable between those with PMDD and the partners (e.g., love: M = 6.19 and 5.82, commitment: M = 6.35 and 6.17 for the partners and those with PMDD respectively). Therefore, it seems likely that the relationship experience is homogenous with regards to the difficulties each party perceives. However, it is important to note that other factors, not measured in the present study, may also influence perceived relationship quality. For example, it is possible that co-occurring mental health conditions in the person with PMDD and/or their partner may be driving these effects. Alternatively, the impact of PMDD on the individual’s ability to work/participate in childcare could lead to additional stressors for both parties and the relationship dynamic as whole. Nonetheless, the medium-large effect sizes reflect the practical implications of our findings and support the need to develop PMDD-specific interventions that support both the person with PMDD and their partner (e.g., PMDD specific relationship guidance). Indeed, these findings suggest that couples may benefit from acknowledging the impact PMDD has had for both parties (i.e., both perceive trust to be an issue in the relationship) and from exploring the ways in which they may work through the perceived issues together.

The samples included in the present study consisted of multiple transgender and non-binary menstruators in the PMDD and non-PMDD group, as well as participants in same sex/queer relationships. This inclusive approach is important for both PMDD research and menstrual cycle research more generally. Firstly, previous research has shown that queer-identified people, especially non-binary and transmasculine menstruators, are often negatively impacted by the societal perception of menstruation (and related concepts, such as PMDD and PMS) as a feminine concept. It has been shown that transgender/non-binary menstruators often engage in “menstrual suppression”, by masking the fact that they have periods or avoiding public restrooms [49], while others report feeling unsafe using their gender-aligned restroom (i.e., male-assigned), due to fear of being “outed” as transgender for using menstrual hygiene products [50]. As such identity concealment is known to have a negative effect on the mental health outcomes of transgender and non-binary individuals [51] it is important for future PMDD research to adopt an inclusive, non-heteronormative approach to recruitment to ensure the experiences of such individuals are acknowledged, and to give societal perceptions of menstruation motivation to adapt in an inclusive manner.

There are limitations that should be considered when interpreting the present findings. Firstly, as the study was conducted using online survey methods, self-selection bias may be present [52]. Specifically, the final sample is likely biased towards people for whom the menstrual cycle is salient; this may explain presence of those with PMS symptoms in the “control” group of Study 1, as opposed to this group being comprised of participants with no menstrual-cycle related symptoms. Secondly, the use of self-report to assess participants cycle phase is a limitation. Indeed, previous studies had to exclude large numbers of participants (up to 46%, [53]) because hormone assays revealed that participants were not in the reported cycle phase. Therefore, the lack of cycle phase effects, and the small effect sizes (e.g., in the relationship quality data of Study 1) may be due to such inaccurate self-reports leading to mislabelling of participant cycle phase (e.g., participants labelled as luteal who may be follicular). Such inaccuracy would likely result in additional variability in the data, in turn masking any true cycle phase effects. It should also be noted that cycle phases were not compared in a pairwise manner in the current study, which may also limit any conclusions about the influence of cycle phase effects. However, it should be noted that cycle phase effects were not the primary variable of interest in this study; nevertheless, future studies of PMDD that wish to investigate the effect of cycle phase should aim to validate cycle phase reports using include direct hormone measures [36]. Thirdly, it should be noted that we did not use a diagnostic process to identify PMDD participants for the present study (e.g., via prospective ratings of symptoms across two menstrual cycles). While this was largely due to the exploratory nature of the study, it limits the interpretation of our results as it is possible that not all participants in our PMDD group would meet the DSM criteria for PMDD. Finally, the cross-sectional design of the present study limits the interpretation of the results to mere associations. That is, we cannot conclusively infer the direction of causality with respect to the association between PMDD and poor relationship/life quality.

However, the findings do pose questions for future research concerning the nature of this relationship. The present findings particularly highlight the need for longitudinal studies to fully understand the nature and direction of the association between PMDD and relationships. Such studies will aid in uncovering the potentially bidirectional relationship between PMDD and relationship quality. For example, it is possible that increased interpersonal conflict within the relationship may trigger or exacerbate PMDD symptoms, heightened symptom severity may trigger or maintain interpersonal conflict, creating a vicious cycle for both parties in the relationship. Thus, longitudinal studies that track symptoms, daily function, and relationship quality across the menstrual cycle are needed to further our understanding of this association, and in turn, inform practical interventions for PMDD couples. Such studies would also allow researchers to precisely determine the possible impact of age as a mediating variable, something which was not possible in the present study. It would also be possible for longitudinal studies to include track participants through the diagnostic process which would ensure that all participants meet the DSM criteria for PMDD and undergo appropriate diagnostic measures (i.e., prospective cycle tracking for at least two months). While such studies would likely require self-reported data, the inclusion of objective measures in a complementary manner to reduce bias (e.g., third-party/clinician observations of symptoms) and error (e.g., using hormone assays in addition to self-reported cycle phase) would be beneficial.

Additional studies would also benefit from adopting a mixed-methods approach, via in-depth interviews or focus groups with those who have PMDD and their partners, to gain further insight into the effects seen in the present study. Specifically, it is not clear why love and commitment scales seem unaffected by PMDD, and there are a range of possible reasons for this. For example, commitment could be driven by genuine commitment to the person/relationship, or it could be underpinned by guilt on the partners’ behalf, and a subsequent reluctance to leave the relationship. Examining the lived experiences of partners in this way is vital to develop a more comprehensive understanding of the impact that PMDD may have on partners themselves, and the relationship dynamic. Moreover, such studies could also aid in identifying possible mediators and moderators regarding the impact of PMDD on partners; given the lack of professional support, it is likely that partners have developed their own support systems and coping mechanisms, which may provide valuable insight for the development of professional support and PMDD-specific interventions.

In conclusion, the present study demonstrated that perceived quality of life was lower in most domains assessed in both those with PMDD and their partners, compared to controls. Relationship quality was also lower for those with PMDD and their partners, for all domains assessed except love and commitment. Taken together, the present studies highlight the need for PMDD-specific support beyond the management of symptoms for the affected individual. Specifically, the findings concerning quality of life in those with PMDD suggest that community-based support and family-focused support may be of benefit. However, at present, non-pharmacological treatments for PMDD lack an evidence base, and further research is needed to appropriately inform the design of such interventions. The present findings also highlight the support needs of the partners of those with PMDD. The development of such support (e.g., PMDD-specific relationship guidance, peer support groups) is important as partners often taken on the role of an informal carer for the person with PMDD, and as such, support for the partners wellbeing will likely have a positive impact on the person with PMDD.

## References

[1] Alevizou F, Vousoura E, Leonardou A. Premenstrual dysphoric disorder: a critical review of its phenomenology, etiology, treatment and clinical status. Curr Womens Health Rev. 2017;14(1):59–66.

[2] Braverman PK. Premenstrual syndrome and premenstrual dysphoric disorder. J Pediatr Adolesc Gynecol. 2007;20(1):3–12. doi: 10.1016/j.jpag.2006.10.007 17289510

[3] Prasad D, Wollenhaupt-Aguiar B, Kidd KN, de Azevedo Cardoso T, Frey BN. Suicidal risk in women with premenstrual syndrome and premenstrual dysphoric disorder: a systematic review and meta-analysis. J Womens Health (Larchmt). 2021;30(12):1693–707. doi: 10.1089/jwh.2021.0185 34415776 PMC8721500

[4] Hamilton J, Parry B, Alagna S, Blumenthal S, Herz E. Premenstrual mood changes - a guide to evaluation and treatment. Psychiatr Ann. 1984;14(6):426–35.

[5] Eisenlohr-Moul TA, Girdler SS, Schmalenberger KM, Dawson DN, Surana P, Johnson JL, et al. Toward the reliable diagnosis of DSM-5 premenstrual dysphoric disorder: the Carolina Premenstrual Assessment Scoring System (C-PASS). Am J Psychiatry. 2017;174(1):51–9. doi: 10.1176/appi.ajp.2016.15121510 27523500 PMC5205545

[6] Reilly TJ, Patel S, Unachukwu IC, Knox C-L, Wilson CA, Craig MC, et al. The prevalence of premenstrual dysphoric disorder: systematic review and meta-analysis. J Affect Disord. 2024;349:534–40. doi: 10.1016/j.jad.2024.01.066 38199397

[7] Eisenlohr-Moul T. Premenstrual disorders: a primer and research agenda for psychologists. Clin Psychol. 2019;72(1):5–17. 32362679 PMC7193982

[8] Halbreich U, Borenstein J, Pearlstein T, Kahn LS. The prevalence, impairment, impact, and burden of premenstrual dysphoric disorder (PMS/PMDD). Psychoneuroendocrinology. 2003;28 Suppl 3:1–23. doi: 10.1016/s0306-4530(03)00098-2 12892987

[9] Pearlstein T. Depressive disorders: premenstrual dysphoric disorder. Psychiatry. 2015:966–80. doi: 10.1002/9781118753378.ch51

[10] Ryu A, Kim T-H. Premenstrual syndrome: a mini review. Maturitas. 2015;82(4):436–40. doi: 10.1016/j.maturitas.2015.08.010 26351143

[11] Yamada K, Kamagata E. Reduction of quality-adjusted life years (QALYs) in patients with premenstrual dysphoric disorder (PMDD). Qual Life Res. 2017;26(11):3069–73. doi: 10.1007/s11136-017-1642-1 28674766

[12] Borenstein JE, Dean BB, Endicott J, Wong J, Brown C, Dickerson V, et al. Health and economic impact of the premenstrual syndrome. J Reprod Med. 2003;48(7):515–24. 12953326

[13] Dean BB, Borenstein JE. A prospective assessment investigating the relationship between work productivity and impairment with premenstrual syndrome. J Occup Environ Med. 2004;46(7):649–56. doi: 10.1097/01.jom.0000131796.62115.84 15247803

[14] Hylan TR, Sundell K, Judge R. The impact of premenstrual symptomatology on functioning and treatment-seeking behavior: experience from the United States, United Kingdom, and France. J Womens Health Gend Based Med. 1999;8(8):1043–52. doi: 10.1089/jwh.1.1999.8.1043 10565662

[15] Robinson RL, Swindle RW. Premenstrual symptom severity: Impact on social functioning and treatment-seeking behaviors. Vol. 9. Journal of Women’s Health & Gender-Based Medicine. US: Mary Ann Liebert, Inc.; 2000. p. 757–68. 10.1089/15246090050147736 11025868

[16] Wittchen H-U, Becker E, Lieb R, Krause P. Prevalence, incidence and stability of premenstrual dysphoric disorder in the community. Psychol Med. 2002;32(1):119–32. doi: 10.1017/s0033291701004925 11883723

[17] Jain A, Rathi P, Reddy S, Kotadia H. Premenstrual syndrome and premenstrual dysphoric disorder among medical and paramedical students - prevalence, pattern and functional impairment. Eur J Mol Clin Med. 2022;09(07):3755–60.

[18] Thakrar P, Bhukar K, Oswal R. Premenstrual dysphoric disorder: Prevalence, quality of life and disability due to illness among medical and paramedical students. J Affect Disord Rep. 2021;4.

[19] Frank B, Dixon D, Grosz H. Conjoint monitoring of symptoms of premenstrual syndrome: impact on marital satisfaction. J Counsel Psychol. 1993;40(1):109–14.

[20] Ryser R, Feinauer LL. Premenstrual syndrome and the marital relationship. Am J Family Ther. 1992;20(2):179–90. doi: 10.1080/01926189208250887

[21] Holt-Lunstad J, Birmingham W, Jones BQ. Is there something unique about marriage? The relative impact of marital status, relationship quality, and network social support on ambulatory blood pressure and mental health. Ann Behav Med. 2008;35(2):239–44. doi: 10.1007/s12160-008-9018-y 18347896

[22] Pieh C, O Rourke T, Budimir S, Probst T. Relationship quality and mental health during COVID-19 lockdown. PLoS One. 2020;15(9):e0238906. doi: 10.1371/journal.pone.0238906 32915878 PMC7485771

[23] Pearlstein T, Steiner M. Premenstrual dysphoric disorder: burden of illness and treatment update. J Psychiatry Neurosci. 2008;33(4):291–301. 18592027 PMC2440788

[24] Bailey JM, Regan TW, Bartlem KM, Wiggers JH, Wye PM, Bowman JA. A survey of the prevalence of modifiable health risk behaviours among carers of people with a mental illness. BMC Public Health. 2019;19(1):1240. doi: 10.1186/s12889-019-7577-4 31500598 PMC6734289

[25] Martín J, Padierna A, van Wijngaarden B, Aguirre U, Anton A, Muñoz P, et al. Caregivers consequences of care among patients with eating disorders, depression or schizophrenia. BMC Psychiatry. 2015;15:124. doi: 10.1186/s12888-015-0507-9 26054966 PMC4459460

[26] Stansfeld S, Smuk M, Onwumere J, Clark C, Pike C, McManus S, et al. Stressors and common mental disorder in informal carers--an analysis of the English Adult Psychiatric Morbidity Survey 2007. Soc Sci Med. 2014;120:190–8. doi: 10.1016/j.socscimed.2014.09.025 25259657 PMC4224501

[27] van Wijngaarden B, Koeter M, Knapp M, Tansella M, Thornicroft G, Vázquez-Barquero J-L, et al. Caring for people with depression or with schizophrenia: are the consequences different? Psychiatry Res. 2009;169(1):62–9. doi: 10.1016/j.psychres.2008.06.013 19625087

[28] Byrom NC. Supporting a friend, housemate or partner with mental health difficulties: the student experience. Early Interv Psychiatry. 2019;13(2):202–7. doi: 10.1111/eip.12462 28707357

[29] Smallwood J, Jolley S, Makhijani J, Grice S, O’Donoghue E, Bendon P, et al. Implementing specialist psychological support for caregivers in psychosis services: a preliminary report. Psychosis. 2016;9(2):119–28. doi: 10.1080/17522439.2016.1259647

[30] Yesufu-Udechuku A, Harrison B, Mayo-Wilson E, Young N, Woodhams P, Shiers D, et al. Interventions to improve the experience of caring for people with severe mental illness: systematic review and meta-analysis. Br J Psychiatry. 2015;206(4):268–74. doi: 10.1192/bjp.bp.114.147561 25833867

[31] Zegwaard MI, Aartsen MJ, Grypdonck MHF, Cuijpers P. Mental health nurses’ support to caregivers of older adults with severe mental illness: a qualitative study. BMC Nurs. 2015;14:37. doi: 10.1186/s12912-015-0087-5 26109909 PMC4479244

[32] Sin J, Murrells T, Spain D, Norman I, Henderson C. Wellbeing, mental health knowledge and caregiving experiences of siblings of people with psychosis, compared to their peers and parents: an exploratory study. Soc Psychiatry Psychiatr Epidemiol. 2016;51(9):1247–55. doi: 10.1007/s00127-016-1222-7 27121259 PMC5025483

[33] Sin J, Norman I. Psychoeducational interventions for family members of people with schizophrenia: a mixed-method systematic review. J Clin Psychiatry. 2013;74(12). 10.4088/JCP.12r08308 24434103

[34] Osborn E, Wittkowski A, Brooks J, Briggs PE, O’Brien PMS. Women’s experiences of receiving a diagnosis of premenstrual dysphoric disorder: a qualitative investigation. BMC Womens Health. 2020;20(1):242. doi: 10.1186/s12905-020-01100-8 33115437 PMC7594422

[35] Wang Y-X, Arvizu M, Rich-Edwards JW, Stuart JJ, Manson JE, Missmer SA, et al. Menstrual cycle regularity and length across the reproductive lifespan and risk of premature mortality: prospective cohort study. BMJ. 2020;371:m3464. doi: 10.1136/bmj.m3464 32998909 PMC7526082

[36] Schmalenberger KM, Tauseef HA, Barone JC, Owens SA, Lieberman L, Jarczok MN, et al. How to study the menstrual cycle: practical tools and recommendations. Psychoneuroendocrinology. 2021;123:104895. doi: 10.1016/j.psyneuen.2020.104895 33113391 PMC8363181

[37] Steiner M, Macdougall M, Brown E. The premenstrual symptoms screening tool (PSST) for clinicians. Arch Womens Ment Health. 2003;6(3):203–9. doi: 10.1007/s00737-003-0018-4 12920618

[38] Harper A, Power M, Orley J, Herrman H, Schofield H, Murphy B, et al. Development of the World Health Organization WHOQOL-BREF Quality of Life Assessment. Psychol Med. 1998;28(3):551–8. doi: 10.1017/s0033291798006667 9626712

[39] Fletcher GJ, Simpson JA, Thomas G. The measurement of perceived relationship quality components: a confirmatory factor analytic approach. Personal Soc Psychol Bull. 2000;26(3):340–54.

[40] Joseph S, Becker S, Elwick H, Silburn R. Adult carers quality of life questionnaire (AC-QoL): development of an evidence-based tool. Mental Health Rev J. 2012;17(2):57–69. doi: 10.1108/13619321211270380

[41] Miklowitz D, Chung B. Family-focused therapy for bipolar disorder: reflections on 30 years of research. Fam Process. 2016;55(3):483–99. 27471058 10.1111/famp.12237 PMC5922774

[42] Berry E, Davies M, Dempster M. Exploring the effectiveness of couples interventions for adults living with a chronic physical illness: a systematic review. Patient Educ Couns. 2017;100(7):1287–303. doi: 10.1016/j.pec.2017.02.015 28228340

[43] Elwick H, Joseph S, Becker S, Becker F. Manual for the Adult Carer Quality of Life Questionnaire (AC-QoL). The Princess Royal Trust for Carers. London, UK. 2010.

[44] Joo J, Bone L, Forte J, Kirley E, Lynch T, Aboumatar H. The benefits and challenges of established peer support programmes for patients, informal caregivers, and healthcare providers. Family Pract. 2022;39(5):903–12. 10.1093/fampra/cmac004 PMC9508871 35104847

[45] Vaughan C, Trail TE, Mahmud A, Dellva S, Tanielian T, Friedman E. Informal caregivers’ experiences and perceptions of a web-based peer support network: mixed-methods study. J Med Internet Res. 2018;20(8):e257. doi: 10.2196/jmir.9895 30154074 PMC6134228

[46] Broadbent P, Thomson R, Kopasker D, McCartney G, Meier P, Richiardi M, et al. The public health implications of the cost-of-living crisis: outlining mechanisms and modelling consequences. Lancet Reg Health Eur. 2023;27:100585. doi: 10.1016/j.lanepe.2023.100585 37035237 PMC10068020

[47] Campbell L, Stanton S. Adult attachment and trust in romantic relationships. Curr Opin Psychol. 2019;25(1):148–51. 30096516 10.1016/j.copsyc.2018.08.004

[48] Demorest AP. Happiness, love, and compassion as antidotes for anxiety. J Posit Psychol. 2020;15:438–47.

[49] Chrisler JC, Gorman JA, Manion J, Murgo M, Barney A, Adams-Clark A, et al. Queer periods: attitudes toward and experiences with menstruation in the masculine of centre and transgender community. Cult Health Sex. 2016;18(11):1238–50. doi: 10.1080/13691058.2016.1182645 27212580

[50] Lane B, Perez-Brumer A, Parker R, Sprong A, Sommer M. Improving menstrual equity in the USA: perspectives from trans and non-binary people assigned female at birth and health care providers. Cult Health Sex. 2022;24(10):1408–22. doi: 10.1080/13691058.2021.1957151 34365908 PMC9912750

[51] Rood BA, Maroney MR, Puckett JA, Berman AK, Reisner SL, Pantalone DW. Identity concealment in transgender adults: A qualitative assessment of minority stress and gender affirmation. Am J Orthopsychiatry. 2017;87(6):704–13. doi: 10.1037/ort0000303 29154610

[52] Schonlau M, Couper MP. Options for Conducting Web Surveys. Statist Sci. 2017;32(2). doi: 10.1214/16-sts597

[53] Gordon HW, Corbin ED, Lee PA. Changes in specialized cognitive function following changes in hormone levels. Cortex. 1986;22(3):399–415. doi: 10.1016/s0010-9452(86)80004-1 3533421

